# Sport fans’ curiosity and impulsive buying: mediation of social media use intensity

**DOI:** 10.3389/fspor.2025.1519003

**Published:** 2025-02-10

**Authors:** Chen-Yueh Chen, Ya-Lun Chou, Yi-Hsiu Lin, Yen-Kuang Lin

**Affiliations:** ^1^ Doctoral Program for Transnational Sport Management and Innovation, College of Management, National Taiwan Sport University (NTSU), Taoyuan, Taiwan; ^2^ Master Program of Sport Facility Management and Health Promotion, National Taiwan University, Taipei, Taiwan; ^3^ Graduate Institute of Athletics and Coaching Science, College of Athletics, National Taiwan Sport University, Taoyuan, Taiwan

**Keywords:** sports fans’ curiosity, social media use intensity, impulsive buying, mediation analysis, SEM

## Abstract

**Introduction:**

Sports fans' curiosity and impulsive buying tendencies are important topics in sports marketing, yet the mediating role of social media use intensity in linking these variables remains underexplored. Grounded in the Stimulus-Organism-Response (S-O-R) theory, this study examines how social media use intensity mediates the relationship between sports fans' curiosity and impulsive buying behavior.

**Methods:**

The study sampled 623 Taiwanese sports fans, including baseball and basketball enthusiasts, to investigate these relationships. Structural Equation Modeling (SEM) was employed to test the proposed hypotheses, focusing on the mediating effect of social media use intensity.

**Results:**

The results indicate that social media use intensity fully mediates the relationship between sports fans' curiosity and impulsive buying tendencies. This highlights the significant role of digital engagement in shaping consumer behavior among sports fans.

**Discussion:**

These findings emphasize the importance of fostering social media engagement as a strategic tool in sports marketing. By transforming fans' curiosity into tangible purchasing behavior, this study provides valuable theoretical and practical contributions to understanding fan behavior and offers actionable recommendations for sports marketers seeking to enhance their marketing strategies in the digital era.

## 1 Introduction

In the current marketing environment, satisfying consumer needs is a core strategy for brand success (1). Within the field of sports marketing, sports fans' purchasing behaviors not only underscore the significance of the sports industry market but also create business opportunities for companies and sponsors (2). Sports fans' emotional investment in teams, athletes, and related products highlights the importance of understanding their needs and motivations. Among these factors, curiosity is considered a key driver of fan behavior (3).

Wann and James (4) identified curiosity as an intrinsic motivation that encourages sports fans to explore new products or experiences. Highly curious fans are typically more engaged in exploratory behaviors, as curiosity possesses both cognitive drive and an element of mystery. Brands often leverage a “mystique appeal” to stimulate consumer curiosity, thereby fostering emotional engagement and purchasing behaviors (5). However, existing literature predominantly focuses on general consumer contexts, leaving a gap in understanding the role of curiosity in sports fan behavior (3, 6). Thus, exploring the relationship between sports fans' curiosity and their purchasing decisions, particularly in the context of impulsive buying, holds significant practical value.

Curiosity not only motivates sports fans to explore sports-related information but also drives consumption behavior and the intention to engage in emerging sports, particularly through social media platforms (7). Moreover, research indicates that when consumer curiosity is triggered, it can lead to immediate and unplanned consumption behaviors to satisfy that curiosity. For instance, fans may purchase team-related merchandise or participate in new sports experiences (8). This immediacy positions curiosity as a critical driver of impulsive purchasing, especially under the influence of sports events or athletes, where purchase impulses are more pronounced (9).

Furthermore, the interactivity and immediacy of social media provide additional opportunities for emotional connection and enhanced brand loyalty, thereby fostering purchasing behaviors (10). Social media enables sports fans to interact with brands and explore content, as they spend significant time on these platforms receiving information and engaging with fellow fans (11). When sports fans' curiosity is stimulated, they actively seek information related to athletes or teams on social media, reinforcing their interaction with brands (12). Daume and Hüttl-Maack (13) found a significant relationship between the intensity of social media use and consumer behavioral tendencies, particularly in emotion-driven purchasing decisions, where social media usage notably increases the likelihood of impulsive buying.

Although existing studies have explored the impact of social media on consumer behavior (14, 15), there remains a lack of in-depth research on how sports fans' curiosity, mediated through social media usage intensity, influences impulsive buying behavior. Prior research highlights the influential role of curiosity on consumer purchasing behaviors, particularly when curiosity drives consumers to explore brand or product information (13). However, most studies have concentrated on general consumer contexts. Mastromartino and Zhang (16) emphasized the need to better understand the purchasing behaviors of sports fans under conditions of high emotional investment, representing a notable research gap.

This study, based on the Stimulus-Organism-Response (S-O-R) model, is the first to conceptualize sports fans' curiosity as a stimulus variable, exploring its effect on social media usage intensity (organism) and further on impulsive buying tendencies (response). The results demonstrate that the S-O-R model has substantial explanatory power in digital marketing and real-time interactive contexts, particularly when emotional and psychological factors drive consumer behaviors (17). Lou et al. (18) further support this assumption, noting that social media's real-time interactivity enhances fans' brand loyalty and emotional engagement, thereby validating this study's core hypotheses.

This research employs structural equation modeling (SEM) to analyze how sports fans' curiosity influences impulsive buying behavior through the intensity of social media use. The findings not only address a theoretical gap in the sports marketing literature but also provide practical implications for digital marketing strategies among sports brands. Based on the study results, sports brands can design innovative marketing campaigns that stimulate fan curiosity and leverage social media interactivity to strengthen emotional connections, thereby enhancing purchase intentions and actual consumption behaviors. These strategies are particularly relevant to sports marketing, helping marketers attract sports fans, boost brand loyalty, and increase market competitiveness.

## 2 Literature review and hypothesis development

### 2.1 S-O-R theory

This study applies the Stimulus-Organism-Response (S-O-R) model to systematically explore how sports fans' curiosity influences impulsive buying tendencies through the mediating role of social media usage intensity. The S-O-R model, proposed by Mehrabian (19), has been widely utilized in the field of consumer behavior to explain the interaction between environmental stimuli (Stimulus), the psychological state of the organism (Organism), and behavioral responses (Response) (20). Particularly in highly emotion-driven markets, the S-O-R model effectively illustrates the psychological and behavioral mechanisms of consumers (17).

In the S-O-R model, the “Stimulus” represents key external factors from the environment. This study, for the first time, identifies sports fans' curiosity as a core external stimulus. Curiosity drives sports fans to actively seek information and experiences related to their favorite teams or athletes (21). For instance, empirical studies in sports marketing contexts have shown that moderate information gaps can significantly stimulate sports fans' curiosity, thereby increasing their intention to watch sports events (8). Another study revealed that curiosity encourages individuals with high openness to seek sports-related information, thereby increasing their consumption of sports television and online content (7). Therefore, sports fans' curiosity can clearly serve as a stimulus, enhancing emotional involvement and potentially driving consumer behavior.

The “Organism” refers to the internal psychological and emotional states triggered by the stimulus. In this study, social media usage intensity is considered the core organism variable, representing the cognitive and emotional responses driven by curiosity (18). When sports fans browse match highlights, follow athlete updates, or interact with fellow fans on social media, they not only gain cognitive satisfaction but also experience strong emotional resonance. This further deepens their sense of involvement and loyalty toward brands or products (22). For example, short-form video platforms such as TikTok or Instagram effectively satisfy sports fans' information needs through innovative content formats, fostering frequent usage and interaction (12).

The “Response” refers to the behavioral outcomes resulting from the stimulus and internal psychological changes. This study identifies impulsive buying tendencies among sports fans as the response variable, reflecting the combined effects of curiosity and social media usage intensity (16). For instance, research has shown that when fans closely follow their favorite teams or athletes' social media activities, they are more likely to be influenced by brand promotions or limited-time offers, leading to immediate purchasing behaviors (9, 23). This impulsive buying behavior is particularly prominent in highly emotionally charged situations.

Scacco and Muddiman (24) observed that curiosity drives audiences to actively engage with media by clicking, reading, and sharing content to fulfill their information exploration needs, further reinforcing their interaction with media content. Additionally, Mahama et al. (25) found that curiosity-driven social media usage not only promotes continuous information searching but can also lead to irrational responses, such as excessive browsing or impulsive purchasing in high-intensity usage scenarios. This indicates that curiosity-driven social media usage fulfills information gaps while strengthening individuals' reliance on content, thereby facilitating active participation and reinforcing behavioral outcomes. Consequently, curiosity not only stimulates individuals' exploratory behaviors toward media content but also influences their responses through social media usage intensity, becoming a critical driver in consumer behavior.

Based on the Stimulus-Organism-Response (S-O-R) model, this study hypothesizes that sports fans' curiosity serves as the “Stimulus,” which, through the mediating role of social media usage intensity (“Organism”), influences their impulsive buying tendencies (“Response”). The effectiveness of this model has been validated in numerous studies focusing on consumer behavior and emotion-driven markets (26, 27). By integrating sports fans' behavioral characteristics with the S-O-R framework, this study enriches the model's application in the field of sports marketing and provides a novel perspective for understanding curiosity-driven behavioral mechanisms.

### 2.2 Curiosity of sports fans

Curiosity is a major driving force in human behavior, motivating individuals to explore unfamiliar environments and seek new information (28, 29). Curiosity is typically categorized into two types: diversive curiosity and specific curiosity (30). Diversive curiosity refers to a general desire for novelty, driving individuals to seek new stimuli or experiences, while specific curiosity is associated with solving a particular problem or filling a knowledge gap, prompting individuals to seek targeted information (31). These types of curiosity may impact consumers' decision-making processes, especially in high-engagement consumption contexts (13).

In the context of sports marketing, sports fans' curiosity serves as a significant driver of their behaviors. When fans have strong curiosity about information related to teams or athletes, they are more likely to actively seek out this information and become emotionally invested (32). This curiosity extends beyond an interest in games and athlete performances to include attention to related products and brands. Empirical findings suggest that a moderate knowledge gap can significantly enhance fans' curiosity, which, in turn, strengthens their engagement and viewing intentions for new sports products (8).

Additionally, research by (33) indicates that sports fans' curiosity is closely linked to behavioral outcomes. They found that timely updates and diverse content effectively satisfy fans' curiosity about games and athletes, enhancing brand identification and purchase intentions. Watanabe et al. (34) also argue that this emotional connection leads fans to actively participate in brand-related activities, potentially increasing purchasing behaviors. Consequently, social media has become a primary means for sports fans to explore and satisfy their curiosity. Marketers can leverage this by enhancing interaction with fans and creating engaging content that stimulates consumption behaviors.

### 2.3 Sports fans' curiosity and impulsive buying

Previous research highlights curiosity as a key psychological mechanism driving consumer behavior, particularly in facilitating unplanned purchasing actions. Curiosity can motivate individuals to actively seek new information and experiences, with this desire for the unknown prompting exploratory behavior that increases the likelihood of purchase when consumers encounter new products or information (13). Hill et al. (35) indicate that when consumers' curiosity is triggered, they often experience an impulse to take immediate action. This impulse is typically accompanied by a high level of emotional engagement, leading to impulsive buying, as curiosity fosters a highly engaged psychological state, making consumers more susceptible to external stimuli. Especially in digital marketing contexts, curiosity-driven consumer behavior can accelerate purchasing decisions when product information is presented enticingly (36).

In the context of sports marketing, Park et al. (2015) show that a moderate knowledge gap can enhance sports fans' curiosity, strengthening their engagement and viewing intentions for new sports products, making it a crucial driver of product or event engagement. For instance, when advertisements create a degree of ambiguity, sports fans tend to feel heightened curiosity, which fosters a positive emotional response, encouraging them to engage more actively and increasing their attention to the product. This ambiguity can lead to an inclination toward impulsive buying tendencys (37). Based on these theoretical and empirical insights, this study proposes H1: Sports fans' curiosity positively predicts impulsive buying tendency.

### 2.4 Sports fans' curiosity and social media use intensity

Curiosity is a powerful psychological driver, motivating individuals to continuously seek new information to fulfill their need for the unknown (29). For sports fans, curiosity often drives them to actively search for and explore sports-related content on social platforms. Social media, in particular, offers real-time game updates, insights into athletes' lives, and discussions surrounding events, which serve as primary sources of curiosity-driven engagement (38). Additionally, a strong link may exist between fans' high curiosity and frequent social media use, as these platforms not only provide a wealth of information but also enable interaction with other fans. This continual access to information satisfies fans' curiosity, enhancing their sense of engagement and involvement (18). This high level of interactivity and immediacy allows fans to engage with others across various platforms, contributing to increased usage intensity. During peak sports seasons, fans tend to use social media frequently to receive real-time updates on games and share and discuss this information with others (22).

Thus, based on sports fans' desire for information and the interactive, real-time feedback mechanisms provided by social media, it can be inferred that fans' curiosity may significantly boost their Social Media Use Intensity. Social media effectively meets fans' information needs and strengthens their engagement, leading to increased platform usage. Based on the above discussion, this study proposes H2: Sports fans' curiosity positively predicts Social Media Use Intensity.

### 2.5 Social media use intensity and impulsive buying

Social media plays a crucial role in the daily lives of sports fans, offering real-time information, interactive platforms, and close connections with athletes and teams (39). Studies show that when consumers spend extended periods on social media, they become more susceptible to advertisements, promotional content, and others' purchasing behaviors, which can lead to impulsive buying (40, 41). For sports fans, social media updates not only satisfy their interest in games and athletes but may also stimulate their purchasing desires. When social media use becomes habitual, fans may engage in less rational decision-making (42).

Moreover, social media's high interactivity allows sports fans to participate in discussions and activities in real-time, further reinforcing their emotional investment and brand loyalty (43). Thus, this study posits that increased Social Media Use Intensity among sports fans may elevate the likelihood of impulsive buying. The immediacy and interactivity of social media not only increase fans' exposure to brands but also amplify their emotional engagement and desire to purchase. Based on this reasoning, this study proposes H3: Social Media Use Intensity positively predicts impulsive buying tendency among sports fans.

## 3 Methodology and measures

### 3.1 Sampling and data collection

This study was approved by the Ethics Review Committee at National Taiwan University. The study targets fans of Taiwan's professional baseball (CPBL) and professional basketball (P. LEAGUE+ and T1 League) leagues.

Firstly, professional baseball and basketball in Taiwan have experienced rapid growth in recent years, with a large fan base. Since its establishment in 1990, the Chinese Professional Baseball League (CPBL) has become a vital part of Taiwan's sports culture, attracting a vast number of dedicated fans who follow team updates. Meanwhile, the P. LEAGUE+ and T1 League have risen in popularity, particularly among younger sports fans who actively engage in online interactions and frequently use social media to follow games and player updates. Thus, the fan bases of these two leagues are suitable samples for this study. Additionally, Taiwanese baseball and basketball fans exhibit high levels of interactivity and emotional investment, making them a significant group for examining consumer behavior. The emotional investment and loyalty of sports fans are closely related to their engagement, especially when interacting with teams and players on social media, where such interactions often strengthen their attachment. Given the extensive marketing activities within Taiwan's professional sports on social media platforms, these fan groups are ideal for exploring the relationship between Social Media Use Intensity and purchasing behavior.

Furthermore, selecting fans of Taiwanese baseball and basketball also aids in understanding fan behavior patterns within the Asian sports market. The professional sports market in Asia is expanding rapidly, driven by the growing demand for sporting events and related products among fans. This provides a rich context for studying consumer behavior. Therefore, choosing Taiwanese baseball and basketball fans allows for an in-depth exploration of the relationships among sports fans' curiosity, Social Media Use Intensity, and impulsive buying tendency, providing empirical support for research in sports marketing and consumer behavior.

### 3.2 Measurements

This study utilized an online survey to assess sports fans' curiosity, social media use intensity, and impulsive buying tendencies. Online surveys were chosen for their advantages in overcoming geographical limitations, reducing time and costs, and facilitating rapid response collection. Participants were recruited through convenience sampling, considering that Taiwanese baseball and basketball fans frequently use Facebook and Line to discuss games and related content. These platforms were selected to effectively capture sports fans with varying media usage habits, thereby reflecting the diversity of social media behaviors.

Prior to completing the survey, participants were screened to confirm their eligibility as baseball or basketball fans. Qualified participants then proceeded to the formal survey, with ample time provided to ensure they could complete the questionnaire comfortably. To enhance response rates, participants were offered an incentive, allowing them to enter a raffle for shopping vouchers upon survey completion by providing their email addresses.

The questionnaire design included key variables such as sports fans' curiosity, social media use intensity, and impulsive buying tendencies, along with basic demographic information. All variables were measured using established scales from previous literature, modified to align with the context of this study. Specifically, the curiosity scale was adapted from Park et al. (32), the social media use intensity scale from Ellison et al. (44), and the impulsive buying tendency scale from Darrat et al. (45). Each item was rated on a 5-point Likert scale, ranging from 1 (“strongly disagree”) to 5 (“strongly agree”). The questionnaire emphasized clarity and simplicity, avoiding ambiguity to ensure the reliability and validity of the measurement tools.

Furthermore, to enhance the accuracy and robustness of the findings, data were collected and separately analyzed for baseball fans and basketball fans. This approach facilitated a meaningful comparison of fan behaviors across different sports contexts, providing greater granularity and strengthening the study's conclusions.

## 4 Results

### 4.1 Demographic and measurement model results

Descriptive statistics reveal that the sample consists of 61% male and 39% female respondents, indicating a predominance of male interest in baseball, with this trend even more pronounced among basketball fans. The age distribution shows a significant proportion of young adults, reflecting a stronger interest in Taiwanese professional baseball and basketball among younger demographics. In terms of income, most fans fall within the NT$20,001 to NT$60,000 range, suggesting a notable purchasing capacity among fans, providing valuable insights for marketers. Additional demographic details are presented in Table 1.

**Table 1:** Summary on demographic variables.

Variable	*N*_1_ *=* 345; *N*_2_ = 282	
	*N*	Percent (%)
Gander		
Male	210 (251)	61% (90%)
Female	135 (27)	39% (10%)
Age		
20–30	217 (214)	63% (77%)
31–40	88 (58)	26% (21%)
41–50	36 (6)	10% (2%)
51–60	4 (0)	1% (0%)
Income (TWD)		
<20,000	103 (107)	30% (38%)
20,001–40,000	116 (87)	34% (31%)
40,001–60,000	80 (61)	23% (22%)
60,001–80,000	25 (18)	7% (6%)
80,001–100,000	7 (2)	2% (1%)
>100,001	14 (3)	4% (1%)
Education		
High school or below	29 (13)	9% (5%)
Junior college or undergraduate	230 (206)	66% (74%)
Postgraduate or above	86 (59)	25% (21%)

N1, Sample size for baseball; N2, sample size for basketball. The numbers presented outside (inside) the parentheses refer to the baseball (basketball).

The reliability and validity analysis indicates high internal consistency within the model. First, the Cronbach's *α* values for all variables range from .92 to .95, suggesting satisfactory internal consistency. Second, confirmatory factor analysis shows that all relevant indicators fall within acceptable ranges (Table 2). Additionally, based on (46) criterion, the average variance extracted (AVE) for each construct is greater than the squared correlations with other constructs, with all AVE values exceeding 0.5, confirming discriminant validity (Table 3). Finally, Harman's single-factor test and principal component analysis detect no significant common method bias, with the variance explained by baseball and basketball fan samples at 40% and 41%, respectively, both below the 50% threshold for total variance.

**Table 2:** Baseball and basketball sports CFA results.

Factor/item	*N*_1_ = 345; *N*_2_ = 278			
	*M*	SD	*λ*	*T*
Sport's fans Curiosity (SC), [*α* = .93 (.92), AVE = .53 (.50), CR = .92 (.92)]				
1. I often spend time examining statistics about my favorite team.	3.28 (3.54)	.93 (.1.00)	.65 (.57)	– (–)
2. I enjoy discussing new sport players, teams, games, and events with friends.	3.59 (3.65)	.87 (.80)	.75 (.79)	12.17* (9.82*)
3. When I miss games, I often search for the final results on television, the Internet, and/or in newspaper.	3.88 (3.69)	.92 (.99)	.71 (.59)	11.62* (8.05*)
4. I enjoy reading articles about new athletes, teams, games, and events.	3.73 (3.84)	.96 (.91)	.80 (.74)	12.77* (9.42*)
5. I often think about why my favorite team's strategy to beat a rival team.	3.49 (3.62)	.93 (.88)	.90 (.89)	14.00* (10.49*)
6. I am curious about sports.	3.52 (3.67)	.90 (.86)	.90 (.90)	13.92* (10.52*)
7. I want to know more about sports.	3.52 (3.81)	.93 (.91)	.71 (68)	11.58* (8.95*)
8. I am intrigued by what is happening in sports.	3.34 (3.53)	.84 (.96)	.69 (.74)	11.31* (9.39*)
9. I would enjoy visiting a sporting goods factory related to my favorite sport to see how their products are made.	3.41 (3.35)	.92 (1.02)	.67 (.74)	11.05* (9.41*)
10. Figuring out how much it would cost to construct a brand new stadium interests me.	3.43 (3.21)	.97 (1.04)	.66 (.67)	10.97* (8.80*)
11. I am curious about how big a sport stadium is.	3.67 (3.10)	.96 (1.09)	.57 (.55)	9.60* (7.65*)
Social media intensity (SMI), [α = .93 (.95), AVE = .70 (.75), CR = .93 (.95)]				
1.1. Social media is part of my everyday activity	3.19 (2.72)	1.38 (1.37)	.85 (.87)	– (–)
2. I am proud to tell people I'm onSocial media	3.15 (3.37)	1.20 (1.27)	.75 (.79)	16.39* (16.85*)
3. Social media has become part of my daily routine	3.40 (3.18)	1.28 (1.39)	.88 (.87)	21.29* (20.12*)
4. I feel out of touch when I haven't logged ontoSocial media for a while	3.57 (3.16)	1.20 (1.36)	.86 (.88)	20.72* (20.38*)
5. I feel I am part of the Social media community	2.81 (2.70)	1.26 (1.37)	.82 (.87)	18.90* (20.03*)
6. I would be sorry if Social media shut down	3.38 (3.09)	1.27 (1.34)	.84 (.89)	19.29* (21.00*)
Impulsive buying (IB), [α = .92 (.93), AVE = .78 (.83), CR = .92 (.93)]				
1. “Just do it” describes the way I buy things	2.00 (1.96)	1.07 (1.07)	.87 (.87)	– (–)
2. I often buy things without thinking	1.82 (1.83)	1.01 (1.06)	.90 (.94)	22.20* (22.47*)
3. “I see it, I buy it” describes me	1.88 (1.91)	1.06 (1.15)	.89 (.92)	21.80* (21.53*)

CFA, confirmatory factor analysis; α, Cronbach's alpha coefficient; AVE, average variance extracted; λ, standardized factor loading; *t*, *t*-value; –, reference parameter.

*χ*^2^/df =510.168/167 = 3.05 (596.982/167 = 3.57); goodness of fit (GFI) = .86 (.80); SRMR = .05 (.07); root mean square error of approximation (RMSEA) = .07 (.09); normed fit index (NFI) = .90 (.87); non-normed fit index (NNFI) = .92 (.89); comparative fit index (CFI) = .93 (.90). The numbers presented outside (inside) the parentheses refer to the baseball (basketball).

* *p* < .001.

**Table 3:** Summary for discriminant validity.

	*N*_1_ = 345; *N*_2_ = 278		
	SC	SMI	IB
SC	**.53 (.50)**		
SMI	.15* (.18*)	**.70 (.75)**	
IB	.04* (.03*)	.10* (.08*)	**.78 (.83)**

The bold numbers along the diagonal represent the AVE values.

Numbers listed along the diagonal denote the AVE values. Numbers listed in the lower and upper triangles refer to the shared variances between constructs in baseball and basketball, respectively. The numbers presented outside (inside) the parentheses refer to the baseball (basketball).

* *p* < .001.

### 4.2 Structural equation modeling (SEM) analysis results

**Figure 1:**
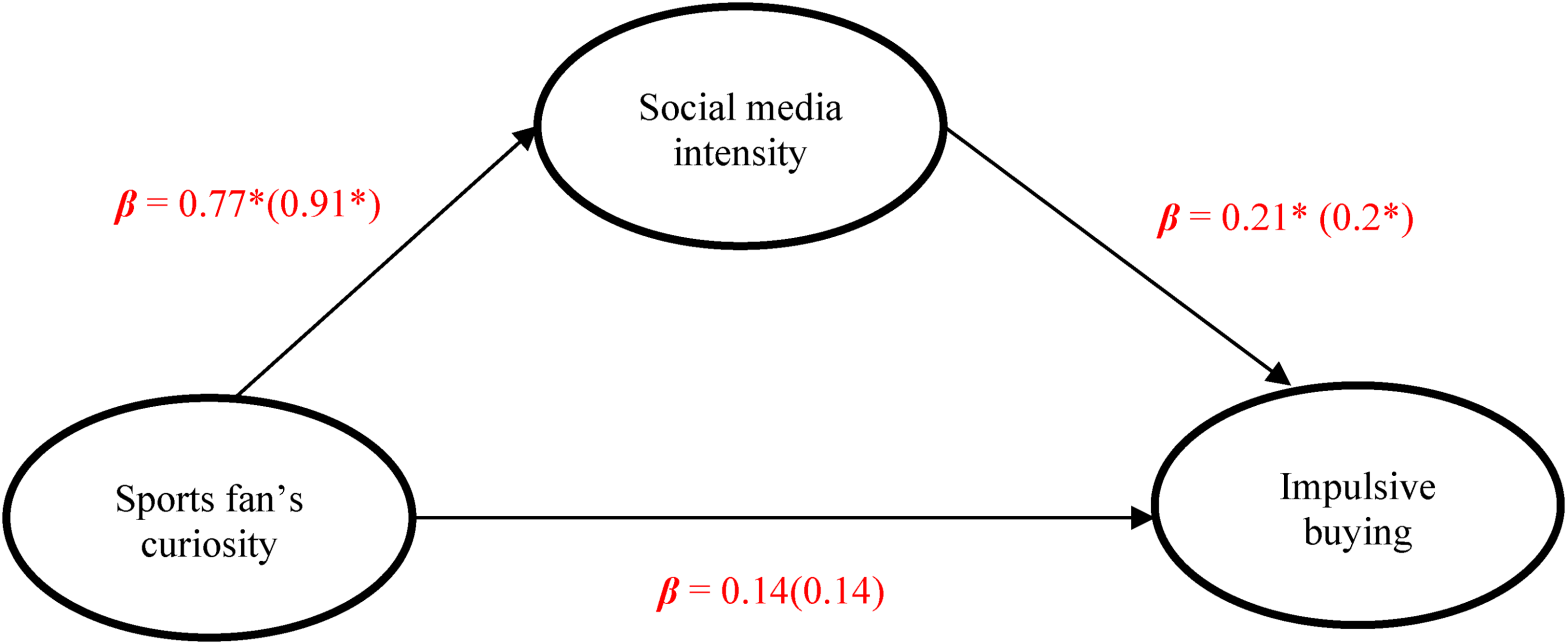
Mediating effects of social media use between sports fans curiosity and impulsive buying, numbers presented outside (inside) the parentheses refer to the baseball (basketball). *p < .001.

Structural Equation Modeling (SEM) analysis was conducted using R-4.4.2 (Table 4). A total of 623 participants were recruited for this study, comprising 345 baseball fans and 278 basketball fans. In accordance with SEM analysis requirements, the sample size met the recommended ratio of 10–15 observations per parameter (47), ensuring the representativeness of the results. After confirming the absence of outliers, the analysis proceeded. The model fit indices indicate a good model fit, and the path analysis reveals that all paths, except for the direct path between sports fans' curiosity and impulsive buying, are significant. This finding suggests that the relationship between sports fans' curiosity and impulsive buying is significantly positive only when mediated by Social Media Use Intensity, thus providing partial support for the hypotheses (Figure 1).

**Table 4:** Structural model results.

Path	*N*_1_ = 345, *N*_2_ = 278		
	*Β*	*t*	Results
SC → IB	0.14 (0.14)	1.49 (1.20)	H1 Not supported
SC → SMI	0.77* (0.91*)	6.50 (6.11)	H2 supported
SMI → IB	0.21* (0.20*)	4.52 (3.67)	H3 supported

Goodness of fit indexes structural model tested; *χ*^2^/df = 510.168/167 = 3.05 (596.982/167 = 3.57); GFI = .86(.80); SRMR = .05 (.07); RMSEA = .08(.09); NFI = .90 (.87); TLI = .92 (.89); IFI = .93 (.90); CFI = .93 (.90).

The numbers presented outside (inside) the parentheses refer to the baseball (basketball).

* *p* *<* *.*001.

Specifically, sports fans' curiosity does not have a significant direct positive relationship with impulsive buying (*β* = 0.14, *p* = 0.14 for baseball fans; *β* = 0.14, *p* = 0.23 for basketball fans). However, sports fans'curiosity is significantly associated with social media use intensity (*β* = 0.77, *p* < .001 for baseball fans; *β* = 0.91, *p* < .001 for basketball fans). Additionally, social media use intensity has a significant positive predictive relationship with impulsive buying (*β* = 0.21, *p* < .001 for baseball fans; *β* = 0.20, *p* < .001 for basketball fans). These findings provide a robust foundation for subsequent hypothesis testing and highlight the interconnected relationships among the variables under investigation.

The determination coefficients (*R*²) for each latent variable in the research model are as follows: Social Media Use Intensity (.11 for baseball fans and .19 for basketball fans) and impulsive buying (.15 for baseball fans and .09 for basketball fans). These values indicate that sports fans' curiosity serves as a strong driving factor, providing valuable insights into Social Media Use Intensity and impulsive buying tendency.

## 5 Discussion

Grounded in the S-O-R theory, this study examines the impact of sports fans' curiosity, Social Media Use Intensity, and impulsive buying, confirming the relationships among these constructs in two samples—fans of Taiwanese professional baseball and basketball.

The results for H1 reveal that sports fans' curiosity does not significantly predict impulsive buying tendencies for either baseball or basketball fans. Therefore, H1 is not supported. This finding contrasts with previous literature, which identifies curiosity as a key driver of consumer behavior, particularly impulsive buying (48). Prior studies have emphasized that curiosity stimulates exploratory behaviors, triggering immediate purchasing reactions (35). However, this study suggests that sports fans' curiosity primarily manifests as an exploration of information related to teams, players, or matches, rather than directly translating into purchasing behaviors. This reflects the emotional nature of sports fan behaviors.

First, sports fans' curiosity may enhance their social media engagement but does not necessarily lead to purchasing behaviors. This is because sports fans' curiosity often stems from emotional needs rather than material desires. According to Self-Determination Theory (SDT), behaviors driven by intrinsic motivation are more persistent and meaningful but tend to satisfy emotional needs, such as excitement over match outcomes or loyalty to a team, rather than shopping desires (12). In the sports context, this study confirms that curiosity primarily motivates fans to seek information about teams, players, and match results—behaviors rooted in emotional engagement that do not directly result in purchases. Second, the distinction between emotional needs and emotional reactions is also crucial in explaining the non-significant relationship between curiosity and impulsive buying. Sports fans' emotional needs are predominantly reflected in their emotional investment toward teams or players, which is unrelated to purchasing behaviors. According to Li et al. (49), emotional reactions are critical in prompting consumers to make immediate purchasing decisions. When curiosity drives sports fans to explore information about their favorite teams or players, the emotional satisfaction they derive is typically related to emotional bonds, not purchasing behavior. This indicates that curiosity among sports fans is more likely to fulfill emotional needs rather than trigger impulsive buying tendencies. Finally, emotional triggers may play a pivotal role in converting curiosity into purchasing behaviors. Daume and Hüttl-Maack (13) highlighted that emotional triggers, such as promotional activities or emotional attachments, significantly amplify the effect of curiosity on impulsive buying. In the sports fan sample, the absence of such emotional triggers may explain why curiosity does not directly translate into purchasing behaviors. Therefore, this finding suggests that incorporating emotional triggers or promotional campaigns into marketing strategies could be more effective in driving purchasing behaviors among sports fans.

For H2, results show that sports fans' curiosity positively predicts Social Media Use Intensity across both samples (baseball and basketball), thereby supporting H2. This finding further validates the applicability of the S-O-R theory in sports marketing contexts, underscoring the pivotal role of curiosity in driving social media engagement. According to the S-O-R framework, external stimuli, such as fans' curiosity, generate internal psychological responses (Social Media Use Intensity), subsequently affecting behavioral outcomes. This study empirically supports this model by examining fans' behavior on social media, emphasizing curiosity's positive influence on digital engagement. Consistent with Abdourazakou and Deng (22), the results confirm that curiosity drives consumers' engagement on social platforms. For fans, curiosity compels them to stay updated with game progress and player activities, making social media their primary platform for satisfying this exploratory need. As Pandita and Vapiwala (38) note, the demand for information among sports fans is particularly high during events, underscoring curiosity's role in enhancing digital interaction frequency and depth. This study further demonstrates that high curiosity among sports fans leads to frequent social media usage, particularly in response to trending topics and real-time updates.

In digital marketing, sports fans' curiosity translates into dependency on social media (11). point out that the real-time interactivity of social media can effectively drive consumer engagement, positioning curiosity as a key factor for increasing social media usage. This study's findings confirm that fans' usage intensity reflects a strong demand for new information, indicating that brands can capture fans' attention through frequent updates and engaging content. Specifically, sports marketers who create dynamic, real-time, and attractive content can effectively fulfill fans' exploratory needs, promoting sustained digital interaction—a strategy that bolsters a brand's influence and competitive edge in digital environments.

The results for H3 indicate that Social Media Use Intensity positively predicts impulsive buying tendency among sports fans in both samples (baseball and basketball), thereby supporting H3. This result further validates the S-O-R theory, suggesting that internal psychological responses (Social Media Use Intensity) stimulate behavioral responses (impulsive buying). It also highlights social media's potential influence in driving instant purchasing decisions (41). The real-time interactivity of social media not only satisfies fans' information needs but also reinforces their buying inclinations in emotionally driven contexts.

Firstly, this result aligns with existing literature. Studies indicate that social media's high interactivity enhances consumers' emotional involvement in online settings, raising the likelihood of impulsive purchases. Chen et al. (40) found a significant association between active social media use and impulsive buying, particularly when consumers frequently engage on social platforms. Emotional responses can intensify and influence purchasing decisions, as verified by this study, showing that sports fans' high Social Media Use Intensity fosters their purchase impulses, especially during games or brand events. Secondly, the findings emphasize the significant influence of real-time interaction on fans. According to Kennedy and Funk (42), frequent social media interaction decreases consumers' behavioral control, increasing the likelihood of impulsive purchases. The high level of interaction among fans not only brings them closer to brand information but may also strengthen emotional investment, making them more susceptible to emotionally driven purchasing decisions. This suggests that sports brands can utilize social media's high interactivity to capture fans' attention and stimulate impulsive buying tendency.

The most critical finding of this study is that Social Media Use Intensity fully mediates the relationship between sports fans' curiosity and impulsive buying tendency, further validating the effectiveness of the S-O-R model. According to the results, sports fans' curiosity primarily drives their engagement with social media platforms, where interactions enhance their attention to matches and player updates. These behaviors strengthen fans' emotional bonds with teams or brands. Moreover, through the highly interactive and real-time nature of social media, sports fans' demand for information is amplified, leading to increased interaction with brands. These interactions extend beyond passive viewing and commenting; they also involve communication with fellow fans, which further enhances purchase intentions. Singh et al. (41) noted that fan participation in social media discussions deepens emotional investment, which not only strengthens brand loyalty but also stimulates impulsive buying behavior. Thus, Social Media Use Intensity serves as an indicator of internal psychological states and plays a pivotal role in sports fan behaviors. Sports fans' curiosity motivates them to seek information about teams, players, or matches, and this exploratory behavior is satisfied through interactions on social media platforms. High-intensity social media usage is not only a means of emotional gratification but also enhances fans' sense of engagement and loyalty toward brands or teams (18), (Park et al., 2015).

Overall, these findings indicate that Social Media Use Intensity fully mediates the relationship between sports fans' curiosity and impulsive buying tendencies, further validating the applicability of the S-O-R theory in sports marketing. According to the S-O-R model, emotion-driven psychological responses (Social Media Use Intensity) serve as the critical link between external stimuli (curiosity) and behavioral responses (impulsive buying). This extends the S-O-R model by offering a new perspective and confirms the validity of fans' curiosity within this framework, making a significant theoretical contribution. While fans' curiosity drives their desire to explore information, it is the heightened Social Media Use Intensity that ultimately facilitates purchasing behavior. Curiosity relies on the immediacy and interactivity of social media to influence consumer behavior. Chen et al. (40) further note that the high interactivity of social media provides emotional support, making consumers more likely to act on purchase impulses when exposed to brand content in real time. These findings reinforce the argument that curiosity, amplified by social media engagement, can lead to impulsive buying tendencies.

These findings hold significant implications for sports marketing. Social media has become a key tool for brands to establish emotional connections and facilitate real-time interactions, particularly in leveraging its dynamic features to transform fans' curiosity into purchasing behavior. Lim et al. (43) emphasized that highly interactive social media content effectively fosters emotional resonance between brands and fans. Marketers can utilize dynamic content or time-limited promotions to maintain fans' sustained attention, especially during periods of heightened emotional engagement, such as major sports events or outstanding player performances, thereby driving purchasing behaviors. Through real-time updates and interactive promotions, brands can effectively convert emotional investment into tangible purchasing actions.

## 6 Conclusion

This study applies the S-O-R theory to examine the relationships among sports fans' curiosity, Social Media Use Intensity, and impulsive buying tendencies. The results reveal that sports fans' curiosity influences impulsive buying behavior through the mediating role of Social Media Use Intensity. Specifically, fans' curiosity drives them to actively engage in social media interactions, where Social Media Use Intensity plays a pivotal mediating role in promoting impulsive buying tendencies.

The findings underscore the critical role of social media in sports marketing, particularly in enhancing fan emotional engagement, strengthening brand loyalty, and increasing purchase intentions. This study suggests that sports brand managers should focus on increasing the interactivity and immediacy of social media content to strengthen fan engagement and facilitate purchasing behaviors.

From a theoretical perspective, the results contribute to consumer behavior research by demonstrating that sports fans' curiosity drives high levels of social media interaction. This highlights that consumers, in emotionally invested contexts, are more susceptible to emotional responses, which can, in turn, trigger purchasing behavior. These findings align with emotional attachment theory, indicating that emotional needs profoundly influence consumer behaviors.

This study has certain limitations. First, the sample consists exclusively of Taiwanese baseball and basketball fans, which may limit the generalizability of the findings. Future research could expand the scope to include fans from different cultural contexts (e.g., European or American sports fans) to examine the applicability of the results in global markets. Additionally, comparative studies across different sports types (e.g., football, tennis) could identify potential behavioral differences among fans. Moreover, future studies could explore the role of individual characteristics (e.g., the Big Five personality traits) in influencing sports fans' purchasing behaviors. Cross-cultural research is also encouraged to validate the applicability of marketing strategies across diverse markets.

## Data Availability

The datasets presented in this study can be found in online repositories. The names of the repository/repositories and accession number(s) can be found in the article/Supplementary Material.
